# Performance comparison of second- and third-generation sequencers using a bacterial genome with two chromosomes

**DOI:** 10.1186/1471-2164-15-699

**Published:** 2014-08-21

**Authors:** Mari Miyamoto, Daisuke Motooka, Kazuyoshi Gotoh, Takamasa Imai, Kazutoshi Yoshitake, Naohisa Goto, Tetsuya Iida, Teruo Yasunaga, Toshihiro Horii, Kazuharu Arakawa, Masahiro Kasahara, Shota Nakamura

**Affiliations:** CLC bio Japan Inc., a QIAGEN Company, 204 Daikanyama Park Side Village, 9-8 Sarugakucho, Shibuya-ku, Tokyo, 150-0033 Japan; Department of Infection Metagenomics, Research Institute for Microbial Diseases, Osaka University, 3-1 Yamadaoka, Suita, Osaka, 565-0871 Japan; Department of Computational Biology, Graduate School of Frontier Sciences, The University of Tokyo, Kashiwa, 277-8561 Japan; Laboratory of DNA Data Analysis, National Institute of Genetics, 1111 Yata, Mishima, Shizuoka, 411-8540 Japan; Institute for Advanced Biosciences, Keio University, 5322 Endo, Fujisawa, Kanagawa, 252-0882 Japan; Department of Bacteriology, Okayama University Graduate School of Medicine, 2-5-1 Kita-ku Shikata-cho, Okayama, 700-8558 Japan

**Keywords:** Next-generation sequencing, *de novo* assembly, Illumina MiSeq, Ion Torrent PGM, Roche 454 GS Junior, PacBio RS system

## Abstract

**Background:**

The availability of diverse second- and third-generation sequencing technologies enables the rapid determination of the sequences of bacterial genomes. However, identifying the sequencing technology most suitable for producing a finished genome with multiple chromosomes remains a challenge. We evaluated the abilities of the following three second-generation sequencers: Roche 454 GS Junior (GS Jr), Life Technologies Ion PGM (Ion PGM), and Illumina MiSeq (MiSeq) and a third-generation sequencer, the Pacific Biosciences RS sequencer (PacBio), by sequencing and assembling the genome of *Vibrio parahaemolyticus*, which consists of a 5-Mb genome comprising two circular chromosomes.

**Results:**

We sequenced the genome of *V. parahaemolyticus* with GS Jr, Ion PGM, MiSeq, and PacBio and performed *de novo* assembly with several genome assemblers. Although GS Jr generated the longest mean read length of 418 bp among the second-generation sequencers, the maximum contig length of the best assembly from GS Jr was 165 kbp, and the number of contigs was 309. Single runs of Ion PGM and MiSeq produced data of considerably greater sequencing coverage, 279× and 1,927×, respectively. The optimized result for Ion PGM contained 61 contigs assembled from reads of 77× coverage, and the longest contig was 895 kbp in size. Those for MiSeq were 34 contigs, 58× coverage, and 733 kbp, respectively. These results suggest that higher coverage depth is unnecessary for a better assembly result. We observed that multiple rRNA coding regions were fragmented in the assemblies from the second-generation sequencers, whereas PacBio generated two exceptionally long contigs of 3,288,561 and 1,875,537 bps, each of which was from a single chromosome, with 73× coverage and mean read length 3,119 bp, allowing us to determine the absolute positions of all rRNA operons.

**Conclusions:**

PacBio outperformed the other sequencers in terms of the length of contigs and reconstructed the greatest portion of the genome, achieving a genome assembly of “finished grade” because of its long reads. It showed the potential to assemble more complex genomes with multiple chromosomes containing more repetitive sequences.

**Electronic supplementary material:**

The online version of this article (doi:10.1186/1471-2164-15-699) contains supplementary material, which is available to authorized users.

## Background

Next-generation sequencing (NGS) technologies have dramatically changed genomic research. NGS instruments, the so-called second-generation sequencers, generate large volumes of data compared with conventional Sanger sequencers. Before 2010, although the cost of reading a whole genome was rapidly decreasing, the use of NGS technologies was still limited to large genome sequencing centers because of technical and logistical difficulties associated with the operation of the instruments and requirements for computer hardware and data analysis. The advent of benchtop sequencers has accelerated sequencing efforts in small centers and laboratories. For example, the 454 GS Junior (GS Jr), released by Roche in early 2010 as the first benchtop sequencer, uses the same emulsion PCR technology [1] as the Roche GS FLX. The Life Technologies Ion PGM (Ion PGM) benchtop sequencer, which was launched at the beginning of 2011, utilizes semiconductor technology [2]. The Illumina MiSeq (MiSeq) benchtop sequencer became available at the end of 2011 and employs the same sequencing-by-synthesis technology [3, 4] as the Illumina GAII and HiSeq sequencers. With the annual emergence of new NGS instruments, experimental procedures such as library preparation and analysis methods require continual improvement.

Second-generation sequencers generate massive amounts of short reads, which differ in throughput and length from reads produced by Sanger sequencers. To assemble massive amounts of short reads, a new type of algorithm using de Bruijn graphs has flourished, as illustrated by a series of genome assemblers including ABySS [5], ALLPATHS-LG [6], Velvet [7, 8], and SOAPdenovo [9]. Although these algorithms [5–9] have been developed to produce high-quality finished-grade genomes, it remains a challenge to assemble long contigs spanning an entire genome. One of the important factors in successfully obtaining finished genomes is resolving repetitive regions scattered across the genome. It is problematic to reconstruct long repetitive regions by assembling reads shorter than the repetitive regions. Paired ends and mate pairs have been used to tackle this problem. Mate pairs improved scaffold length, but the results using mate-pair assembly have usually been far from finished grade [10, 11].

To address this issue, reads longer than repetitive regions may offer a solution to the assembly problem. The recently launched third-generation Pacific Biosciences RS sequencer (PacBio) system [12] generates long reads with a mean length of 4.5 kbp and with randomly distributed sequencing errors. This evolutionary technology demands a new algorithm to process sequence reads because of the different nature of its reads, whose nucleotide-level accuracy is only 85% [12]. Therefore, several algorithms first correct sequencing errors in reads and then assemble the error-corrected reads [13–15]. PacBio has the advantage of generating long reads but at a throughput lower than that of the second-generation sequencers. One of the disadvantages of PacBio is that the initial installation is more expensive than that of benchtop second-generation sequencers (Additional file 1: Table S1). Combining second- and third-generation sequencing data may be an option [13, 16]; however, these hybrid methods offer limited efficiency because they require more labor and consumables costs for additional library preparation.

Given that various sequencing instruments and software are available for genome sequencing and are evolving, selecting the best one or the best combination is difficult. Performance comparisons of NGS instruments, including that of a third-generation sequencer, have been previously published [17–21]; however, considering the rapid improvement of NGS technologies, frequent comparisons are valuable for selecting the platform providing the best results. Therefore, we performed an updated comparison study of second- and third-generation sequencers using the bacterial genome of *Vibrio parahaemolyticus*, consisting of two chromosomes. Because of the presence of two chromosomes with higher copy numbers of rRNA operons than found in other bacteria, it was difficult to finish the genome sequence [21]. In this study, we demonstrated the reconstruction of the *V. parahaemolyticus* genome using current sequencers.

## Results and Discussion

A summary of sequence run data and their assembly results is shown in Table 1, and the distribution of the sequence read quality of each sequencer is shown in Additional file 2: Figure S1. The assembler for each sequencer was selected on the basis of a previous study and our experiences [22]. To evaluate the accuracy of the generated contigs, we compared them with the *V. parahaemolyticus* reference genome [21] using QUAST v2.3 [23]. Table 2 shows the result of the accuracy evaluation.

**Table 1:** **Data statistics for sequence run and assemblies**

Sequencer	GS Jr	Ion PGM	MiSeq	PacBio
Number of reads	115611	4982888	39656630	120230*
Total bp	48285593	1443005019	9953814130	374942687
Coverage	9	279	1927	73
Mean length	418	290	251	3119
**Assembler**	**Newbler**	**Newbler**	**CLC**	**Sprai**
Number of bp used for assembly	48285593	400000107	299809460	374942687
Number of reads used	115611	1380757	1194460	120230*
Coverage	9	77	58	73
Number of contigs	309	61	34	31
Total bases	5053921	5075085	5103771	5298335
Max length	164926	895358	732626	3288561
N50 contig length	30451	392606	431440	3288561

GS Jr, Ion PGM, and MiSeq data are based on a single run. PacBio data are from three cells. The upper part of the table shows read statistics and the lower part shows the statistics of the best assembly. *Number of reads of PacBio is the number of subreads longer than 500 bp.

**Table 2:** **Accuracy of assembled contigs with respect to the reference genome**

Mismatches	GS Jr	Ion PGM	MiSeq	PacBio	PacBio (>1 M bp)
Number of contigs	309	61	34	31	2
Number of mismatches	133	108	230	389	157
Number of indels	824	2853	184	715	698
Indels length	977	3018	241	818	794
Number of mismatches per 100 kbp	2.6	2.1	4.5	7.5	3.0
Number of indels per 100 kbp	16.3	56.2	3.6	13.8	13.5
Number of misassemblies	0	0	1	13	10
Number of relocations	0	0	1	11	10
Number of translocations	0	0	0	1	0
Number of inversions	0	0	0	1	0
Number of misassembled contigs	0	0	1	5	2
Genome coverage (%)	97.844	98.290	98.499	99.999	99.848
Duplication ratio	1.004	1.000	1.003	1.032	1.007

Generated contigs were compared with the reference genome using QUAST v2.3 [23]. The number of indels is the total number of insertions and deletions in the aligned bases. The number of relocations, inversions, and translocations are classified as misassemblies. A relocation is defined as a misassembly in which the left and right flanking sequences both align to the same chromosome on the reference but are either >1 kb apart or overlap by >1 kb. An inversion is a misassembly in which the left and right flanking sequences both align to the same chromosome but on opposite strands. A translocation is a misassembly in which the flanking sequences align on different chromosomes. Genome coverage is the percentage of bases aligned to the reference genome.

### Genome assembly using GS Junior

A single sequencing run of GS Jr yielded 48 Mbp with 115,611 reads, corresponding to 9× coverage of the *V. parahaemolyticus* genome. The mean length of the GS Jr reads was 418 bp. We selected the Newbler assembler [24], which is optimized for Roche 454 chemistry [22, 24]. The Newbler assembly consisted of 309 contigs with maximum length 164,926 bp. The total length of the contigs was 5,053,921 bp. Long reads are usually superior to short reads for the reconstruction of long contigs; however, this fragmented assembly suggested that low-coverage reads are insufficient for building a small number of long contigs.

The generated contigs were evaluated by comparison with the *V. parahaemolyticus* genome. The contig coverage of the *V. parahaemolyticus* genome was 97.844%. The total number of mismatches was 133, and the number of mismatches per 100 kbp was 2.6. The total number of insertions and deletions (indels) was 824, and the number of indels per 100 kbp was 16.3. These higher rates of errors compared with the other sequencers were largely because of the homopolymer error of 454 chemistry [22].

### Genome assembly using Ion PGM

A single run from Ion PGM using the Ion 318 chip generated 1.44 Gbp with 4,982,888 reads. The mean length of the reads was 290 bp. The read coverage of the genome was 279×. We selected Newbler for Ion PGM because it is known to produce longer contigs for Ion PGM as well [22] because of the similarity of its sequencing chemistry to that of Roche 454.

We employed random sampling to reduce the number of input reads [20] and attempted to find the best amount of input data size for assembly [9]. Six sets of 100 inputs were prepared. The size of the inputs in each set was 100, 200, 300, 400, 500, and 600 Mbp, respectively. These sizes correspond to 19×, 39×, 58×, 77×, 96×, and 116× coverage, respectively. The maximum contig length and N50 contig length of all results are shown in Additional file 3: Figure S2. The best subset contained 61 contigs with maximum contig length of 895,358 bp in the 400 Mbp data set (Additional file 3: Figure S2). The number of reads used for the assembly was 1,380,757, corresponding to 77× genome coverage. The N50 contig length was 392,606 bp, and the total length of the contigs was 5,075,085 bp.

Subsequently, the accuracy was evaluated as that for the GS Jr contigs. The contig coverage of the genome was 98.290%. The total number of mismatches was 108, and the number of mismatches per 100 kbp was 2.1. The total number of indels was 2,853, and the number of indels per 100 kbp was 56.2. Homopolymer error has often been reported for Ion PGM [18, 22], and we could confirm this effect in the assembled contigs, as exemplified in Additional file 4: Figure S3(A).

### Genome assembly using MiSeq

A single run of the MiSeq sequencer generated 9.95 Gbp with 39,656,630 reads in pairs. The read coverage of the genome was 1,927×. The mean length of the reads was 251 bp. We used CLC Assembly Cell as the assembler, which is known as a short-read assembler and has been used for a benchmark sequence comparison [22]. We performed random sampling to find the best subset of reads for assembly. The best subset yielded 34 contigs with a maximum contig length of 732,626 bp. The number of reads used for the assembly was 1,194,460, corresponding to 58× genome coverage. The total length of the contigs was 5,103,771 bp and N50 contig length was 431,440 bp.

The contigs contained 230 mismatches in total and 4.5 mismatches per 100 kbp. There were 184 indels in total and 3.6 indels per 100 kbp. MiSeq has a different error profile than Ion PGM. MiSeq errors are known to occur in GGC motifs [25], and we confirmed this error in the generated contigs. The examples of errors are shown in Additional file 4: Figure S3 (B).

### Evaluation of random sampling

We used random sampling for the assembly of Ion PGM and MiSeq data and selected the best subset. For comparison, Additional file 5: Table S2 shows a summary of assemblies generated by the complete set of reads. Assembly using all 279× coverage reads generated by Ion PGM resulted in 502 contigs that were much more fragmented than the 61 contigs using the sampled reads. Likewise, the N50 contig length using all reads is 110,578 bp, a number much smaller than the 392,606 bp obtained with randomly sampled reads. MiSeq generated coverage of 1,927× in a single run and 42 contigs were generated using all reads by a single run of MiSeq, whereas the number of contigs assembled from the sampled reads was 34. These results suggest that an excessive number of reads does not help and can even harm genome assembly. Widely used assemblers do not assume excess coverage, suggesting that the number of reads fed to assemblers should be optimized by random sampling. The optimized sequencing coverage was reported to be <100 [9, 20].

To determine the factors that improve assembly by random sampling, we compared the best subset with the worst. The subset yielding the fewest contigs was considered the best. The best and worst sampled reads were mapped to the reference *V. parahaemolyticus* genome. On a closer examination of the junction regions, where reads from the worst sampled reads were unable to connect contigs (i.e., gaps), we found that the high-quality reads perfectly matching the reference genome were uniformly distributed in the gap regions of the best sampled reads (Additional file 6: Figure S4). In contrast, the distribution of the high-quality reads from the worst sampled reads was not uniform, suggesting that nonuniform coverage causes a disconnection of contigs. Random sampling enables us to generate different combinations of read sets, some of which contain high-quality reads that uniformly span the genome and aid in constructing long contigs. This finding indicates that random sampling would be a simple and effective procedure for finding the optimum coverage and best combination of reads for *de novo* assembly when excess reads are available.

### Genome assembly using PacBio

Three cells of PacBio data yielded 120,230 subreads longer than 500 bp, amounting to 375 Mbp in total and corresponding to 73× coverage of the *V. parahaemolyticus* genome. Several assemblers have been developed for PacBio data. pacBioToCA is a program that corrects sequencing errors using other sequencers’ reads [13] or using PacBio reads themselves. HGAP does not require other sequencers’ reads to correct errors [14]. We employed Sprai [26], a new tool for correcting PacBio sequencing errors without other sequencers’ reads using multiple alignments of raw PacBio reads. The Sprai algorithm and its performance are shown in Additional file 7. The assembly by Sprai generated 31 contigs using three-cell data, showing better assembly performance than that by HGAP. The results are shown in Additional file 8: Table S3 and Additional file 9: Figure S5. The maximum length of the contigs was 3,288,561 bp, and the second longest contig was 1,875,537 bp. The lengths of these two contigs are almost equal to those of the *V. parahaemolyticus* genome chromosomes 1 and 2 (3,288,558 and 1,877,221 bps, respectively). The other 29 contigs were all <21 kbp. The contig length distribution is shown in Additional file 9: Figure S5. The two chromosomes of *V. parahaemolyticus* were reconstructed without gaps by PacBio reads alone, without using reads from other sequencing platforms or jumping libraries.

To further validate these two contigs, we evaluated their accuracy along with all 31 contigs (Table 2). The coverage of all 31 contigs was 99.999%, whereas that of the longest two contigs was 99.848%. The 31 contigs contained a total of 389 mismatches, whereas the longest two contigs contained 157. The number of mismatches per 100 kbp was 7.5 for the 31 contigs and 3.0 for the longest two contigs. The numbers of indels were 715 and 698, and the numbers of indels per 100 kbp were 13.8 and 13.5, respectively. The majority of PacBio sequencing errors were indels, a characteristic known to be a shortcoming of PacBio [27].

### Comparison of assembled contigs

**Figure 1:**
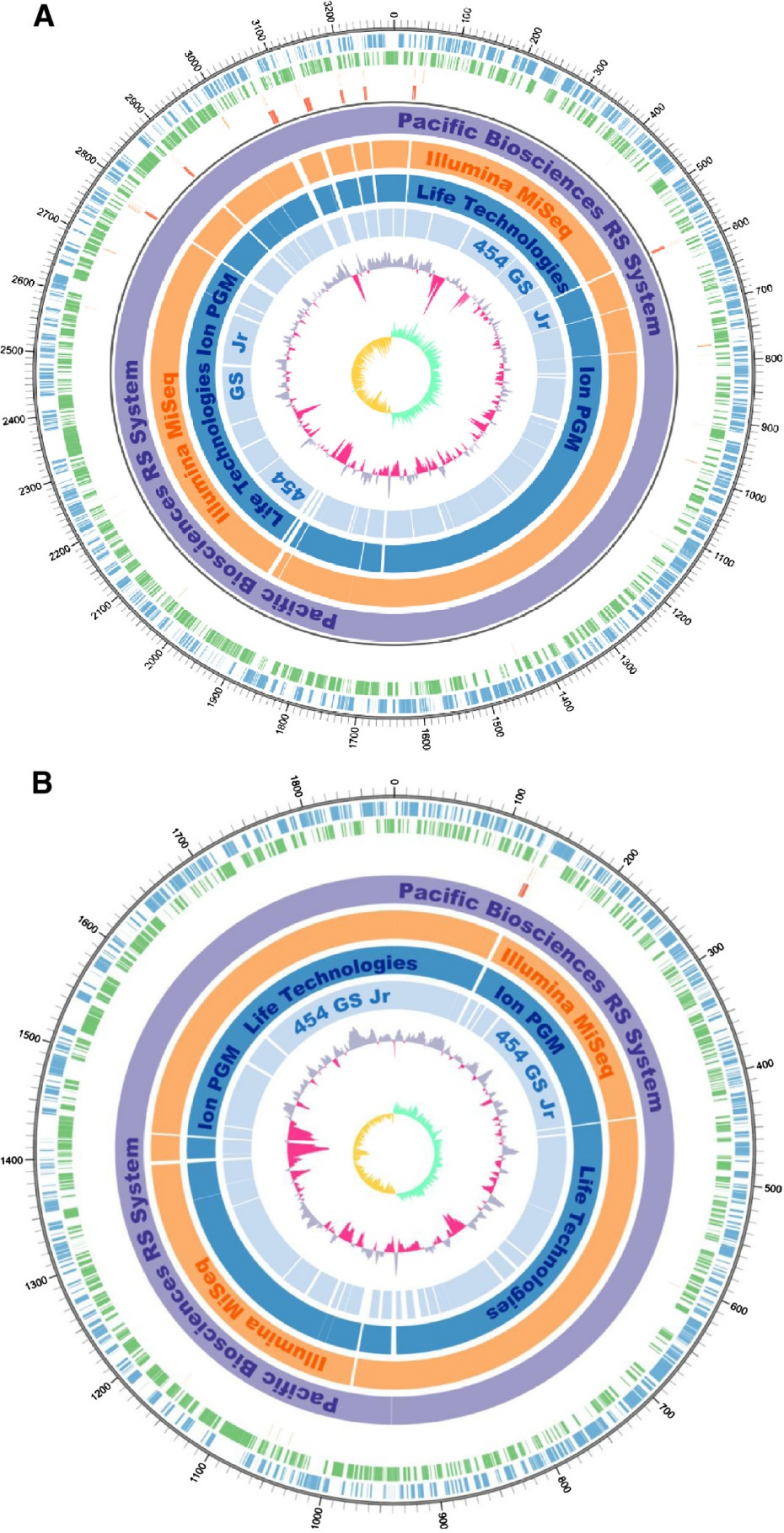
**Contig alignment against the** ***V. parahaemolyticus*** **genome. A** Alignment of contigs to *V. parahaemolyticus* chromosome 1. PacBio, MiSeq, Ion PGM, and GS Jr contigs are aligned to chromosome 1 and visualized with Circos [28]. From outer to inter rings: forward CDS, reverse CDS, tRNA, rRNA, PacBio contigs, MiSeq contigs, Ion PGM contigs, GS Jr contigs, %GC plot, and GC skews. **B** Alignment of contigs to *V. parahaemolyticus* chromosome 2 PacBio, MiSeq, Ion PGM, and GS Jr contigs are aligned to chromosome 2 and visualized using a Circos plot. From outer to inter rings: forward CDS, reverse CDS, tRNA, rRNA, PacBio contigs, MiSeq contigs, Ion PGM contigs, GS Jr contigs, %GC plot, and GC skews.

All contigs from GS Jr, Ion PGM, Miseq, and PacBio were aligned to the *V. parahaemolyticus* genome, as summarized in Figure 1. The contig length distributions are shown in Additional file 10: Figure S6. The sequence assembled using the PacBio sequencer was the highest in quality and genome coverage (Table 2). The Sprai assembler corrected the sequencing errors of PacBio and successfully assembled the reads into two contigs corresponding to the two chromosomes. MiSeq, Ion PGM, and GS Jr all left gaps across contigs. We found that these gaps often fell into rRNA tracts in the genome.

The power of PacBio to generate long reads shows great promise for the assembly of bacterial sequences without hybrid assembly [15, 20]. Previous studies concluded that the accuracy and length of the contigs using PacBio alone surpassed those using second-generation sequencers. However, these studies analyzed bacterial genomes with a single chromosome. In contrast, the present study examined a more complex genome comprising two chromosomes containing 11 copies of rRNA operons. The lengths of 23S rRNA and 16S rRNA sequences are approximately 3.0 kbp and 1.4 kbp, respectively, and the mean read length obtained using PacBio was 3.1 kbp, making it possible to correctly determine the absolute positions of multiple rRNA coding regions (Figure 1). The difficulty of the *V. parahaemolyticus* genome assembly is caused by these rRNA repetitive regions and by similar regions between chromosomes 1 and 2, which may be the cause of misassembly (Additional file 11: Figure S7). These complications made assembly difficult for the second-generation sequencers.

Previously, the *V. parahaemolyticus* genome was sequenced by the Sanger method using multiple libraries with different insert sizes [21]. Libraries with long insert size (4–5 kbp) were used to construct the scaffolds. However, repetitive regions such as rRNA operons required to be independently sequenced to identify the absolute positions. From this experience, we know that jumping libraries would not be useful for accurate reconstruction of the repetitive regions. Long reads that cover not only entire repeat regions but both ends of each repeat region are necessary to determine their absolute positions.

## Conclusions

We compared the abilities of currently available sequencers to assemble a bacterial genome. The use of random sampling improved the assembly of the sequence data from the second-generation sequencers. In the course of upgrading the performance of the second-generation sequencers, the best-subset selection of sequencing data would be more important to make a good assembly of bacterial genome. As described in previous reports [17–21], PacBio achieved a long continuous, finished-grade assembly of a complex bacterial genome. Sequencing technology and chemistry are evolving at a dramatic speed. Future chemistry and instrument updates will bring further improvements, such as support for the sequencing and assembly of higher organisms with multiple chromosomes and the coexistence of multiple genomes in symbiotic organisms. Several challenges in assembling the genomes of higher organisms using PacBio have been published [29–31], although hybrid assembly is required because of the limitations of current PacBio technology including low throughput, high cost, and the amount of DNA required. Our study and these recent challenges reinforce the importance of performing frequent evaluations of the rapidly improving hardware and software for determining genomic sequences.

## Methods

### DNA preparation of the *V. parahaemolyticus*genome

A single colony of *V. parahaemolyticus* (RIMD2210633) from TCBS agar plates was isolated and transferred to 3% NaCl-containing LB medium. Cells were harvested after overnight culture and subjected to PowerSoil DNA Isolation Kit (MO BIO Laboratories). Purified DNA was quantified with a Qubit dsDNA HS Assay kit (Life Technologies). DNA degradation was evaluated by 1% agarose gel electrophoresis using an E-Gel Electrophoresis System (Life Technologies).

### Library preparation, sequencing, and data analysis

#### GS Junior

Genomic DNA (500 ng) was sheared using a GS Rapid Library Prep Nebulizer (Roche) and a library was prepared using a GS Rapid Library Rgt/Adaptors Kit (Roche), according to the manufacturer’s instructions. Sequencing was performed using a GS Junior Titanium Sequencing Kit. The software Newbler v2.5 (Roche) [24] was employed to assemble the 454 GS Junior data with default parameters.

#### Ion PGM

Genomic DNA (2 μg) was sheared using the Covaris S220 (Covaris) and a library was prepared using an Ion Fragment Library Kit (Life Technologies), according to the manufacturer’s instructions. Sequencing was performed using a 318 chip and an Ion PGM Sequencing 400 Kit (Life Technologies). The Ion PGM data were randomly sampled with the sfffile tool v2.5 (Roche) and then assembled with the software Newbler v2.5 (Roche) [24] with default parameters.

#### MiSeq

Genomic DNA (500 ng) was sheared using the Covaris S220 (Covaris) and a library was prepared using ligation-based Illumina multiplex library preparation (LIMprep). Paired end sequencing (250 bp) was performed using a MiSeq v2 500 cycle kit (Illumina). Random sampling and assembly were performed with CLC Assembly Cell v4.10 (CLC bio). Parameters for assembly were bubble size 600 and word size 41.

#### PacBio

Genomic DNA (3 μg) was sheared using the HydroShear Plus (Digilab) and a library was prepared using a DNA Template Prep Kit 2.0 (Pacific Biosciences), according to the manufacturer’s instructions. Sequencing was performed with XL polymerase and a DNA Sequencing Kit C2 (Pacific Biosciences) and three SMRT cells (120 min movies). *De novo* assembly was performed with Sprai v0.9.5 [26] and HGAP v2.1.0 [14] with default parameters. The contigs from Sprai were circularized with a script in the Sprai package when the script detected a significant overlap between the beginning and end of contigs.

#### Evaluation criteria

Contig statistics were used to evaluate the performance. The number of contigs, maximum length of contigs, total length, and N50 contig length were used as general metrics for contig assessment. Contig statistics were calculated with QUAST v2.3 [23].

### Availability of supporting data

The raw sequencing data have been deposited in the DDBJ Sequence Read Archive (DRA) under the accession code DRA002157.

## Electronic supplementary material

Additional file 1: Table S1: Cost and required DNA amount for each sequencer. Sequence cost and DNA requirements for each sequencer. Ion PGM cost is based on an Ion 318 Chip that yields 2 Gb with 400 bp read length. MiSeq information is based on 250 paired-end reads generating 15 Gb. Library preparation information for MiSeq is based on MiSeq Reagent Kit v3. (PDF 89 KB)

Additional file 2: Figure S1: Quality distribution of sequence reads. The mean Phred score and percentage of sequences are plotted on the X- and Y-axes, respectively. All reads were used to generate these graphs. (PDF 952 KB)

Additional file 3: Figure S2: Variations of maximum length and N 50 contig length generated by random sampling. Six sets of 100 random data sets were generated. The size of the inputs in each set was 100 Mbp (19× coverage), 200 Mbp (39×), 300 Mbp (58×), 400 Mbp (77×), 500 Mbp (97×), and 600 Mbp (116×), respectively. (PDF 930 KB)

Additional file 4: Figure S3: Examples of Ion PGM and MiSeq errors. Assembled contigs were aligned to the *V. parahaemolyticus* genome. Mismatches: A) Ion PGM and B) MiSeq. (PDF 887 KB)

Additional file 5: Table S2: Assembly results using all reads. All reads from Ion PGM and MiSeq sequencing were used for *de novo* assembly of six sets. Newbler was used for Ion PGM and CLC Assembly Cell was used for MiSeq assembly. (PDF 29 KB)

Additional file 6: Figure S4: Mapping comparison of best- and worst-sampled reads. The best and worst sampled reads were mapped to the reference *V. parahaemolyticus* genome. The zoomed images show that perfectly matched reads of the best-sampled reads were uniformly distributed in the gap regions of the worst-sampled reads. Mapping was performed with CLC Genomics Workbench v7.0. (PDF 2 MB)

Additional file 7: **Details of the Sprai algorithm and performance validation.** The algorithm of the Sprai and performance benchmarks using the six bacterial genomes in the previous study [15] are shown. (PDF 29 KB)

Additional file 8: Table S3: Comparison between Sprai and HGAP assembly. The number of mismatches was calculated using QUAST v.2.3 [23]. (PDF 46 KB)

Additional file 9: Figure S5: Comparison of the distributions of HGAP and Sprai contigs. The length of the contigs (log10) is plotted on the X-axis and the number of contigs is plotted on the Y-axis. Sprai generated exceptionally long contigs. HGAP [16] generated relatively long contigs but Sprai [26] outperformed HGAP. (PDF 879 KB)

Additional file 10: Figure S6: Distribution of contig sizes. The length of the contigs (log10) is plotted on the X axis and the number of contigs is plotted on the Y axis. The longest PacBio contigs were 3,288,561 and 1,875,537 bps. (PDF 881 KB)

Additional file 11: Figure S7: *V. parahaemolyticus* chromosome alignment. The *V. parahaemolyticus* chromosomes 1 and 2 are aligned by MUMmer (Version 3.22). Minimum length of a match is 10. Forward and reverse complement matches were computed and plot by red and blue respectively. (PDF 220 KB)

## Abbreviations

V. *parahaemolyticus*: Vibrio parahaemolyticus
bp: base pair
GS Jr: Roche 454 GS Junior
Ion PGM: Life Technologies Ion PGM
MiSeq: Illumina MiSeq
PacBio: Pacific Biosciences RS sequencer
NGS: next-generation sequencing
indels: insertions and deletions.

## References

[1] Dressman D, Yan H, Traverso G, Kinzler KW, Vogelstein B (2003). Transforming single DNA molecules into fluorescent magnetic particles for detection and enumeration of genetic variations. Proc Natl Acad Sci.

[2] Rothberg JM, Hinz W, Rearick TM, Schultz J, Mileski W, Davey M, Leamon JH, Johnson K, Milgrew MJ, Edwards M, Hoon J, Simons JF, Marran D, Myers JW, Davidson JF, Branting A, Nobile JR, Puc BP, Light D, Clark TA, Huber M, Branciforte JT, Stoner IB, Cawley SE, Lyons M, Fu Y, Homer N, Sedova M, Miao X, Reed B (2011). An integrated semiconductor device enabling non-optical genome sequencing. Nature.

[3] Bentley DR, Balasubramanian S, Swerdlow HP, Smith GP, Milton J, Brown CG, Hall KP, Evers DJ, Barnes CL, Bignell HR, Boutell JM, Bryant J, Carter RJ, Keira Cheetham R, Cox AJ, Ellis DJ, Flatbush MR, Gormley NA, Humphray SJ, Irving LJ, Karbelashvili MS, Kirk SM, Li H, Liu X, Maisinger KS, Murray LJ, Obradovic B, Ost T, Parkinson ML, Pratt MR (2008). Accurate whole human genome sequencing using reversible terminator chemistry. Nature.

[4] Dohm JC, Lottaz C, Borodina T, Himmelbauer H (2008). Substantial biases in ultra-short read data sets from high-throughput DNA sequencing. Nucleic Acids Res.

[5] Simpson JT, Wong K, Jackman SD, Schein JE, Jones SJM, Birol I (2009). ABySS: a parallel assembler for short read sequence data. Genome Res.

[6] Gnerre S, Maccallum I, Przybylski D, Ribeiro FJ, Burton JN, Walker BJ, Sharpe T, Hall G, Shea TP, Sykes S, Berlin AM, Aird D, Costello M, Daza R, Williams L, Nicol R, Gnirke A, Nusbaum C, Lander ES, Jaffe DB (2011). High-quality draft assemblies of mammalian genomes from massively parallel sequence data. Proc Natl Acad Sci U S A.

[7] Zerbino DR, Birney E (2008). Velvet: algorithms for de novo short read assembly using de Bruijn graphs. Genome Res.

[8] Zerbino DR, McEwen GK, Margulies EH, Birney E (2009). Pebble and rock band: heuristic resolution of repeats and scaffolding in the velvet short-read de novo assembler. PLoS One.

[9] Li R, Zhu H, Ruan J, Qian W, Fang X, Shi Z, Li Y, Li S, Shan G, Kristiansen K, Li S, Yang H, Wang J, Wang J (2010). De novo assembly of human genomes with massively parallel short read sequencing. Genome Res.

[10] Chaisson MJ, Brinza D, Pevzner PA (2009). De novo fragment assembly with short mate-paired reads: Does the read length matter?. Genome Res.

[11] Wetzel J, Kingsford C, Pop M (2011). Assessing the benefits of using mate-pairs to resolve repeats in de novo short-read prokaryotic assemblies. BMC Bioinformatics.

[12] Eid J, Fehr A, Gray J, Luong K, Lyle J, Otto G, Peluso P, Rank D, Baybayan P, Bettman B, Bibillo A, Bjornson K, Chaudhuri B, Christians F, Cicero R, Clark S, Dalal R, Dewinter A, Dixon J, Foquet M, Gaertner A, Hardenbol P, Heiner C, Hester K, Holden D, Kearns G, Kong X, Kuse R, Lacroix Y, Lin S (2009). Real-time DNA sequencing from single polymerase molecules. Science.

[13] Koren S, Schatz MC, Walenz BP, Martin J, Howard JT, Ganapathy G, Wang Z, Rasko DA, McCombie WR, Jarvis ED, Phillippy AM (2012). Hybrid error correction and de novo assembly of single-molecule sequencing reads. Nat Biotechnol.

[14] Chin C-S, Alexander DH, Marks P, Klammer AA, Drake J, Heiner C, Clum A, Copeland A, Huddleston J, Eichler EE, Turner SW, Korlach J (2013). Nonhybrid, finished microbial genome assemblies from long-read SMRT sequencing data. Nat Methods.

[15] Koren S, Harhay GP, Smith TP, Bono JL, Harhay DM, McVey SD, Radune D, Bergman NH, Phillippy AM (2013). Reducing assembly complexity of microbial genomes with single-molecule sequencing. Genome Biol.

[16] Bashir A, Klammer AA, Robins WP, Chin CS, Webster D, Paxinos E, Hsu D, Ashby M, Wang S, Peluso P, Sebra R, Sorenson J, Bullard J, Yen J, Valdovino M, Mollova E, Luong K, Lin S, LaMay B, Joshi A, Rowe L, Frace M, Tarr CL, Turnsek M, Davis BM, Kasarskis A, Mekalanos JJ, Waldor MK, Schadt EE (2012). A hybrid approach for the automated finishing of bacterial genomes. Nat Biotechnol.

[17] Liu L, Li Y, Li S, Hu N, He Y, Pong R, Lin D, Lu L, Law M (2012). Comparison of next-generation sequencing systems. J Biomed Biotechnol.

[18] Quail MA, Smith M, Coupland P, Otto TD, Harris SR, Connor TR, Bertoni A, Swerdlow HP, Gu Y (2012). A tale of three next generation sequencing platforms: comparison of Ion Torrent, Pacific Biosciences and Illumina MiSeq sequencers. BMC Genomics.

[19] Glenn TC (2011). Field guide to next-generation DNA sequencers. Mol Ecol Resour.

[20] Powers JG, Weigman VJ, Shu J, Pufky JM, Cox D, Hurban P (2013). Efficient and accurate whole genome assembly and methylome profiling of E. coli. BMC Genomics.

[21] Makino K, Oshima K, Kurokawa K, Yokoyama K, Uda T, Tagomori K, Iijima Y, Najima M, Nakano M, Yamashita A, Kubota Y, Kimura S, Yasunaga T, Honda T, Shinagawa H, Hattori M, Iida T (2003). Genome sequence of Vibrio parahaemolyticus: a pathogenic mechanism distinct from that of V cholerae. Lancet.

[22] Loman NJ, Misra RV, Dallman TJ, Constantinidou C, Gharbia SE, Wain J, Pallen MJ (2012). Performance comparison of benchtop high-throughput sequencing platforms. Nat Biotechnol.

[23] Gurevich A, Saveliev V, Vyahhi N, Tesler G (2013). QUAST: quality assessment tool for genome assemblies. Bioinformatics.

[24] Margulies M, Egholm M, Altman WE, Attiya S, Bader JS, Bemben LA, Berka J, Braverman MS, Chen Y-J, Chen Z, Dewell SB, Du L, Fierro JM, Gomes XV, Godwin BC, He W, Helgesen S, Ho CH, Irzyk GP, Jando SC, Alenquer MLI, Jarvie TP, Jirage KB, Kim J-B, Knight JR, Lanza JR, Leamon JH, Lefkowitz SM, Lei M, Li J (2005). Genome sequencing in microfabricated high-density picolitre reactors. Nature.

[25] Nakamura K, Oshima T, Morimoto T, Ikeda S, Yoshikawa H, Shiwa Y, Ishikawa S, Linak MC, Hirai A, Takahashi H, Altaf-Ul-Amin M, Ogasawara N, Kanaya S (2011). Sequence-specific error profile of Illumina sequencers. Nucleic Acids Res.

[26] **Sprai** [ http://zombie.cb.k.u-tokyo.ac.jp/sprai/index.html]

[27] Schadt EE, Turner S, Kasarskis A (2010). A window into third-generation sequencing. Hum Mol Genet.

[28] Krzywinski M, Schein J, Birol I, Connors J, Gascoyne R, Horsman D, Jones SJ, Marra MA (2009). Circos: an information esthetic for comparative genomics. Genome Res.

[29] Ganapathy G, Howard JT, Ward JM, Li J, Li B, Li Y, Xiong Y, Zhang Y, Zhou S, Schwartz DC, Schatz M, Aboukhalil R, Fedrigo O, Bukovnik L, Wang T, Wray G, Rasolonjatovo I, Winer R, Knight JR, Koren S, Warren WC, Zhang G, Phillippy AM, Jarvis ED (2014). High-coverage sequencing and annotated assemblies of the budgerigar genome. Gigascience.

[30] Youssef NH, Couger MB, Struchtemeyer CG, Liggenstoffer AS, Prade RA, Najar FZ, Atiyeh HK, Wilkins MR, Elshahed MS (2013). The genome of the anaerobic fungus Orpinomyces sp. strain C1A reveals the unique evolutionary history of a remarkable plant biomass degrader. Appl Environ Microbiol.

[31] Bradnam KR, Fass JN, Alexandrov A, Baranay P, Bechner M, Birol I, Boisvert S, Chapman JA, Chapuis G, Chikhi R, Chitsaz H, Chou W-C, Corbeil J, Del Fabbro C, Docking TR, Durbin R, Earl D, Emrich S, Fedotov P, Fonseca NA, Ganapathy G, Gibbs RA, Gnerre S, Godzaridis E, Goldstein S, Haimel M, Hall G, Haussler D, Hiatt JB, Ho IY (2013). Assemblathon 2: evaluating de novo methods of genome assembly in three vertebrate species. Gigascience.

