# Supplementary material for: Performance comparison of second- and third-generation sequencers using a bacterial genome with two chromosomes
Source: BMC Genomics. 2014 Aug 21;15(1):699. doi: 10.1186/1471-2164-15-699 (PMC4159541; doi:10.1186/1471-2164-15-699)
Supplement: Supplementary file 1 — Additional file 1: Table S1: Cost and required DNA amount for each sequencer. Sequence cost and DNA requirements for each sequencer. Ion PGM cost is based on an Ion 318 Chip that yields 2 Gb with 400 bp read length. MiSeq information is based on 250 paired-end reads generating 15 Gb. Library preparation information for MiSeq is based on MiSeq Reagent Kit v3. (PDF 89 KB) [file 12864_2014_6410_MOESM1_ESM.pdf]

Cost and required DNA comparison

	GS Jr	Ion PGM	MiSeq	PacBio
Instrument cost	$108K	$50K	$99K	$900K
Sequence yield per run	35Mb	2Gb (400bp)	8 Gb	1 Gb/8 SMRT cells
Running cost	$1000/1run(35Mb)	$437/Gb	$93/Gb	$1800/Gb
Sequence Run time	10 hr	7.3 hr	39 hr	16 hr/8 SMRT cells
Other time consuming steps	Library prep: 3hr<br>emPCR: 6hr	Library prep: 3.5 hr<br>emPCR : 8 hr	Library prep: 7 hr	Library prep: 5 hr
DNA requirements	500 ng with 1.8 OD	250 ng	250 ng	100ng (250bp library) - 5µg (20kb library)
