# Supplementary figures and images for: Performance comparison of second- and third-generation sequencers using a bacterial genome with two chromosomes
Source: BMC Genomics. 2014 Aug 21;15(1):699. doi: 10.1186/1471-2164-15-699 (PMC4159541; doi:10.1186/1471-2164-15-699)

Additional file 2, Figure S1

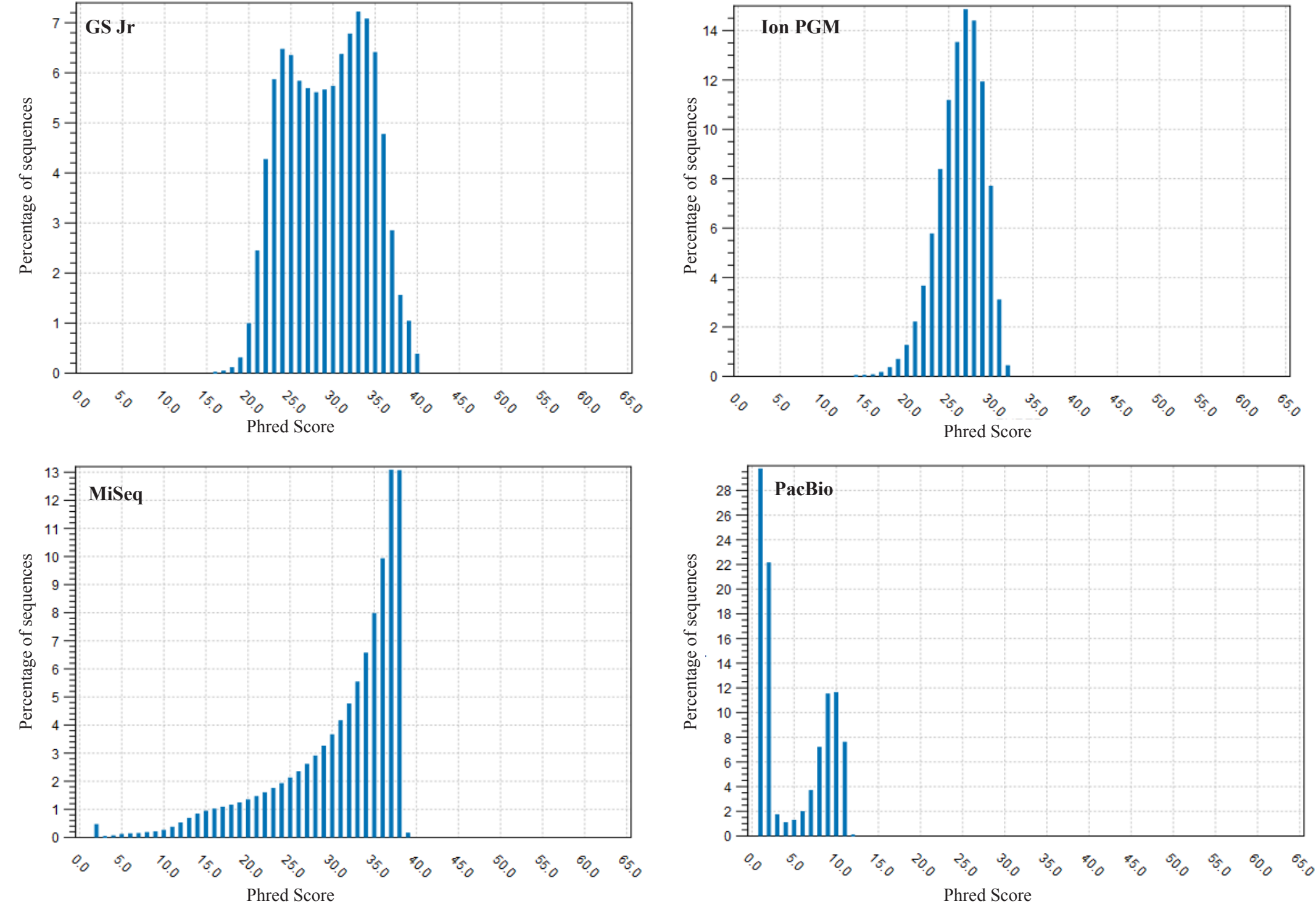

Supplement: Supplementary file 2 — Additional file 2: Figure S1: Quality distribution of sequence reads. The mean Phred score and percentage of sequences are plotted on the X- and Y-axes, respectively. All reads were used to generate these graphs. (PDF 952 KB) [file 12864_2014_6410_MOESM2_ESM.pdf]

Maximum length

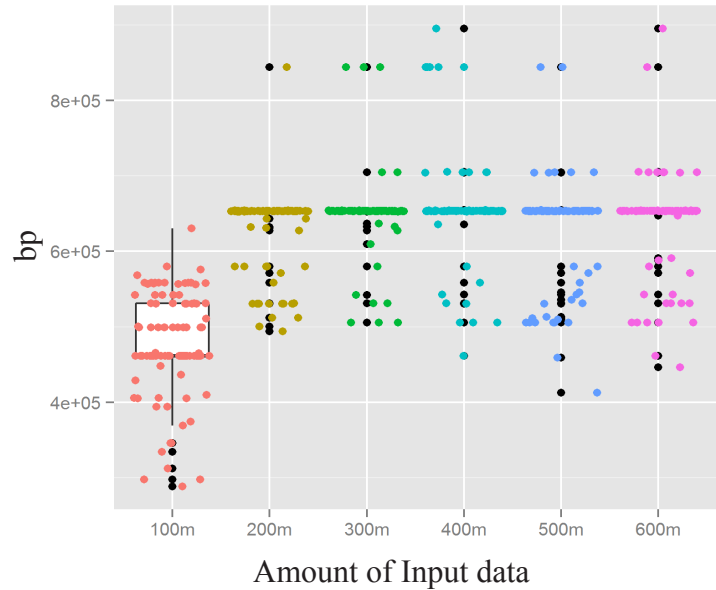

N50 contig length

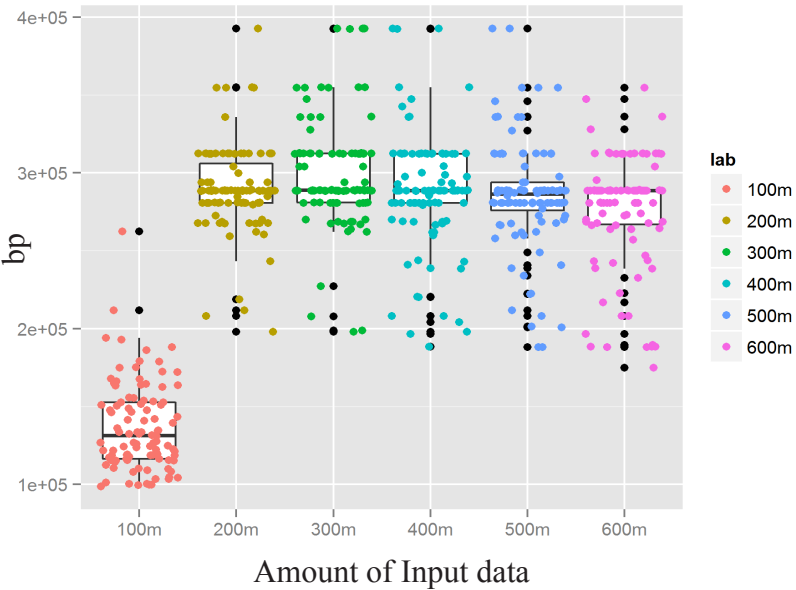

Supplement: Supplementary file 3 — Additional file 3: Figure S2: Variations of maximum length and N 50 contig length generated by random sampling. Six sets of 100 random data sets were generated. The size of the inputs in each set was 100 Mbp (19× coverage), 200 Mbp (39×), 300 Mbp (58×), 400 Mbp (77×), 500 Mbp (97×), and 600 Mbp (116×), respectively. (PDF 930 KB) [file 12864_2014_6410_MOESM3_ESM.pdf]

(A) Ion PGM example error

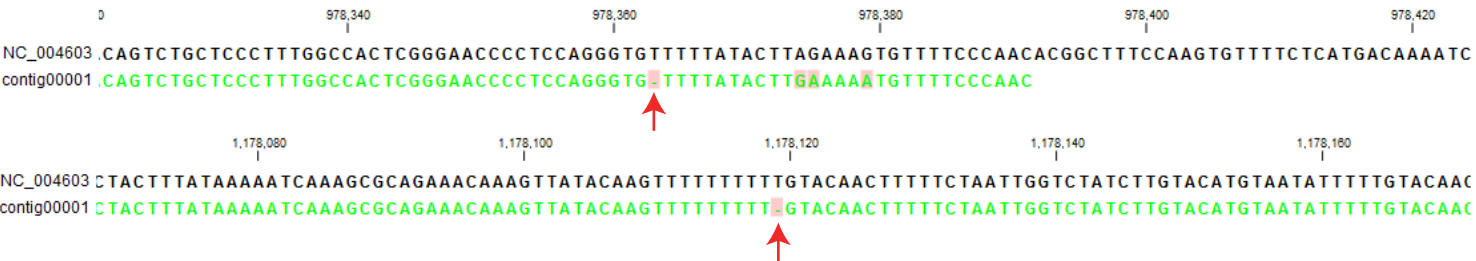

(B) MiSeq example error

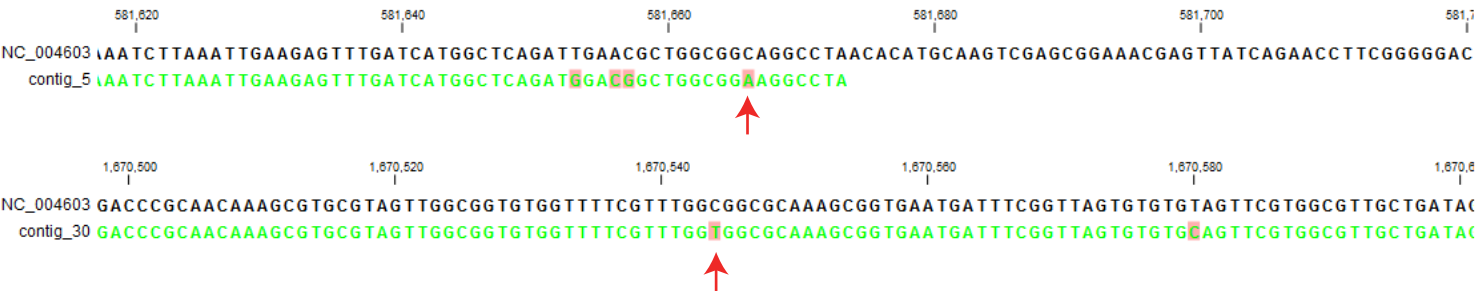

Supplement: Supplementary file 4 — Additional file 4: Figure S3: Examples of Ion PGM and MiSeq errors. Assembled contigs were aligned to the V. parahaemolyticus genome. Mismatches: A) Ion PGM and B) MiSeq. (PDF 887 KB) [file 12864_2014_6410_MOESM4_ESM.pdf]

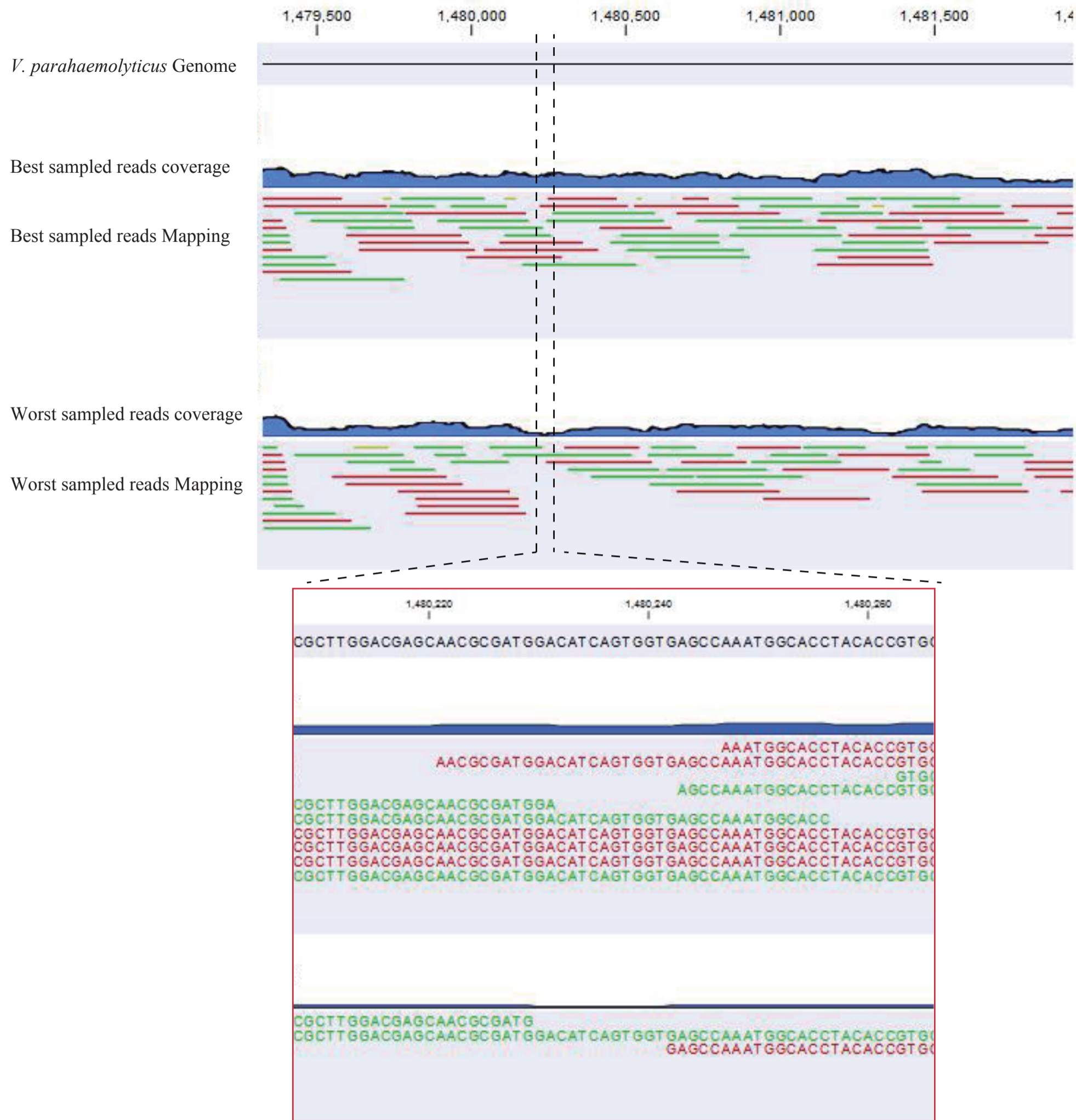

Supplement: Supplementary file 6 — Additional file 6: Figure S4: Mapping comparison of best- and worst-sampled reads. The best and worst sampled reads were mapped to the reference V. parahaemolyticus genome. The zoomed images show that perfectly matched reads of the best-sampled reads were uniformly distributed in the gap regions of the worst-sampled reads. Mapping was performed with CLC Genomics Workbench v7.0. (PDF 2 MB) [file 12864_2014_6410_MOESM6_ESM.pdf]

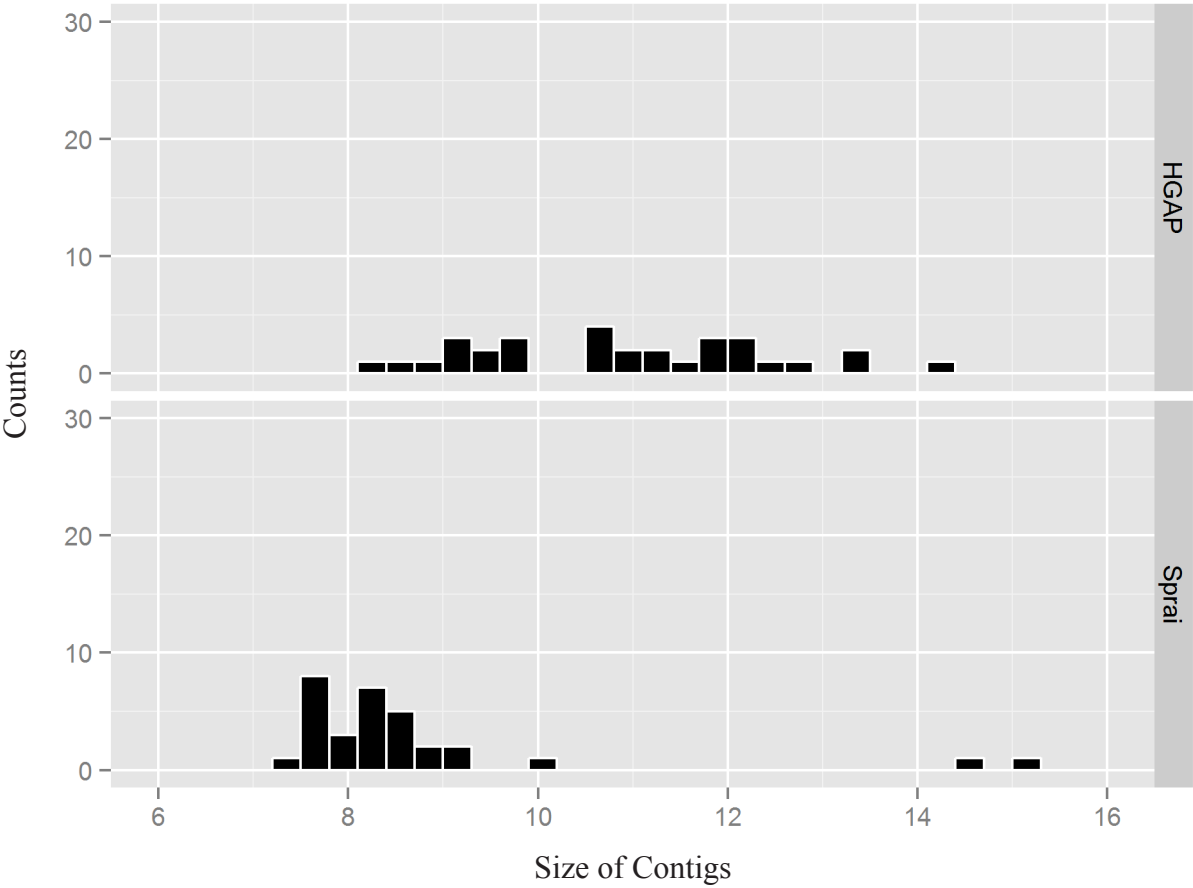

Supplement: Supplementary file 9 — Additional file 9: Figure S5: Comparison of the distributions of HGAP and Sprai contigs. The length of the contigs (log10) is plotted on the X-axis and the number of contigs is plotted on the Y-axis. Sprai generated exceptionally long contigs. HGAP [16] generated relatively long contigs but Sprai [26] outperformed HGAP. (PDF 879 KB) [file 12864_2014_6410_MOESM9_ESM.pdf]

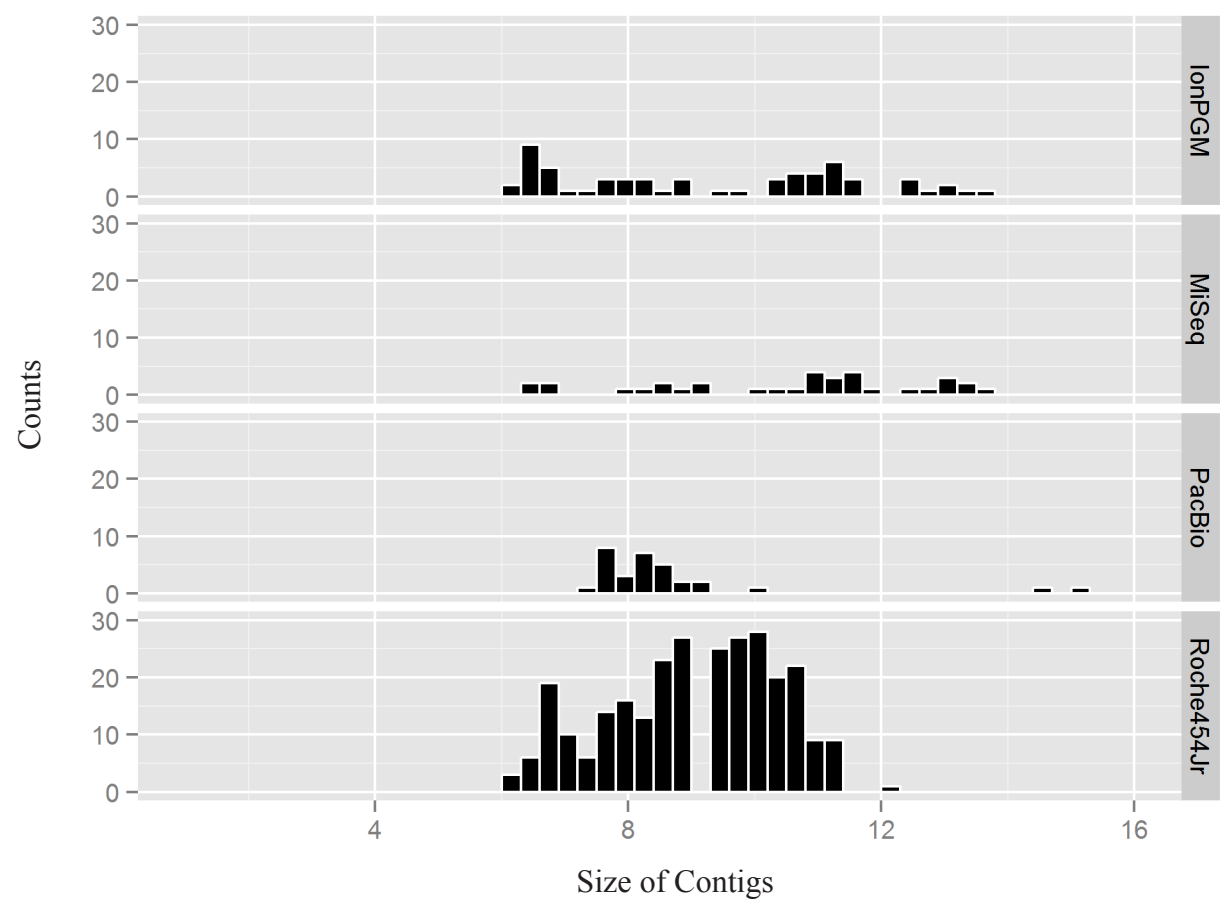

Supplement: Supplementary file 10 — Additional file 10: Figure S6: Distribution of contig sizes. The length of the contigs (log10) is plotted on the X axis and the number of contigs is plotted on the Y axis. The longest PacBio contigs were 3,288,561 and 1,875,537 bps. (PDF 881 KB) [file 12864_2014_6410_MOESM10_ESM.pdf]

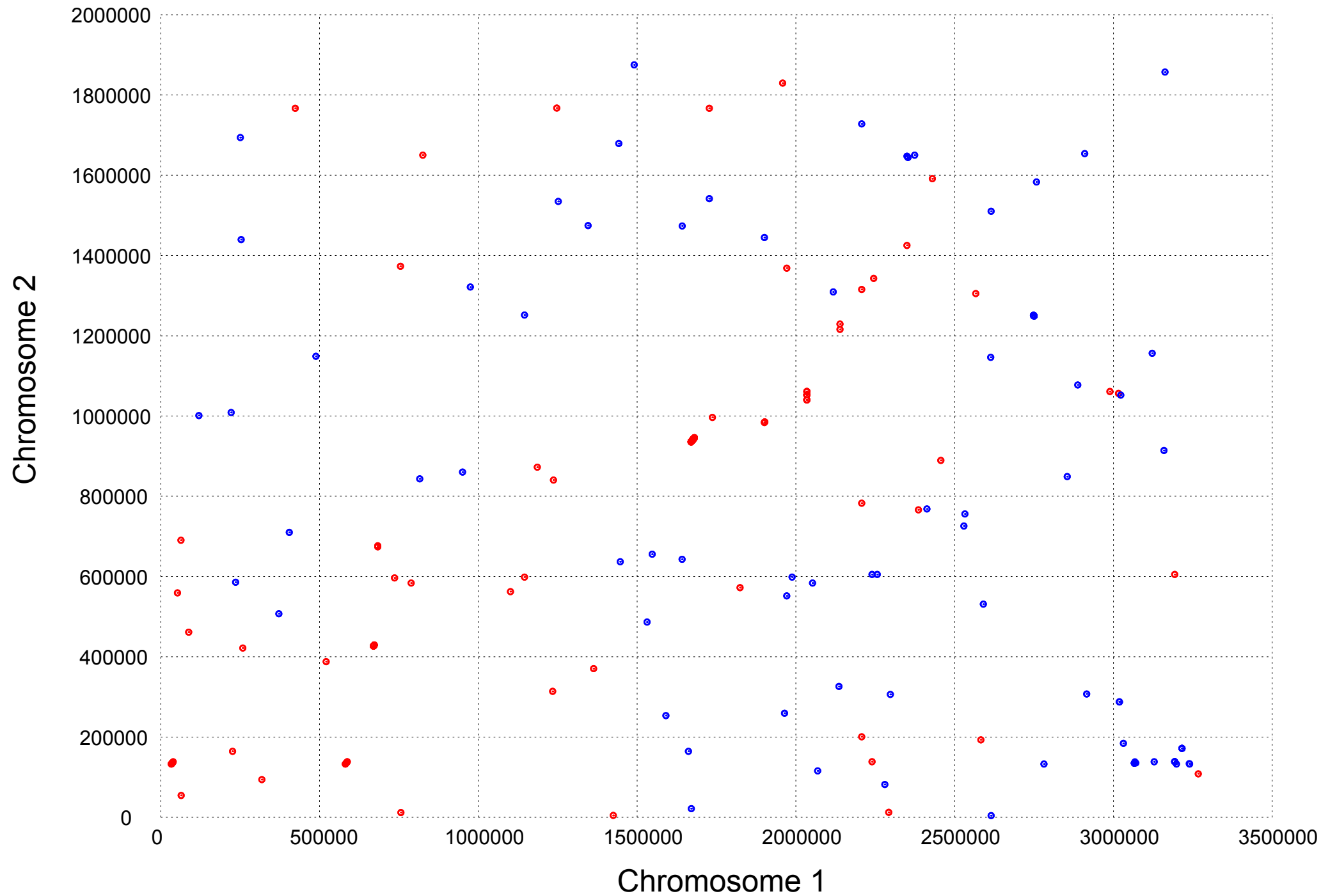

Supplement: Supplementary file 11 — Additional file 11: Figure S7: V. parahaemolyticus chromosome alignment. The V. parahaemolyticus chromosomes 1 and 2 are aligned by MUMmer (Version 3.22). Minimum length of a match is 10. Forward and reverse complement matches were computed and plot by red and blue respectively. (PDF 220 KB) [file 12864_2014_6410_MOESM11_ESM.pdf]
