# Supplementary material for: Performance comparison of second- and third-generation sequencers using a bacterial genome with two chromosomes
Source: BMC Genomics. 2014 Aug 21;15(1):699. doi: 10.1186/1471-2164-15-699 (PMC4159541; doi:10.1186/1471-2164-15-699)
Supplement: Supplementary file 5 — Additional file 5: Table S2: Assembly results using all reads. All reads from Ion PGM and MiSeq sequencing were used for de novo assembly of six sets. Newbler was used for Ion PGM and CLC Assembly Cell was used for MiSeq assembly. (PDF 29 KB) [file 12864_2014_6410_MOESM5_ESM.pdf]

Additional file 5: Table S 2 - Assembly results using all reads

	Number of contigs	N50 contig length(bp)	Max (bp)	Total (bp)
**Ion PGM**	502	110578	653026	5032452
**MiSeq**	42	472546	692574	5112425
