# Supplementary material for: Performance comparison of second- and third-generation sequencers using a bacterial genome with two chromosomes
Source: BMC Genomics. 2014 Aug 21;15(1):699. doi: 10.1186/1471-2164-15-699 (PMC4159541; doi:10.1186/1471-2164-15-699)
Supplement: Supplementary file 7 — Additional file 7: Details of the Sprai algorithm and performance validation. The algorithm of the Sprai and performance benchmarks using the six bacterial genomes in the previous study [15] are shown. (PDF 29 KB) [file 12864_2014_6410_MOESM7_ESM.pdf]

Additional file 7. Overview of the Sprai algorithm and its performance

The detailed algorithm of Sprai will be published elsewhere, but we give an overview of it in this study. Sprai is primarily designed for correcting sequencing errors in single-molecule sequencing reads, and therefore can be integrated with any analysis tools that accept long reads of high accuracy. Among numerous kinds of genome analysis, *de novo* genome assembly is one of the most common analyses, and for this reason we integrated Celera Assembler [1] with Sprai so that Sprai can be used as a *de novo* genome assembler. When the user selects the integration option, Sprai takes raw reads and outputs assembled contigs. Sprai may be used with other genome assemblers, but for now we have tested Sprai only with Celera Assembler.

When Sprai is used as a *de novo* genome assembler, it works very similarly to the HGAP algorithm [2]. Both the algorithms take only Continuous Long Reads (CLRs) from PacBio RS as input and do not require reads from other sequencing platforms such as Illumina HiSeq. They take a set of longer reads in the input raw reads as “seed reads,” and these are error-corrected and assembled with Celera Assembler. They both align the entire set of input reads against the seed reads and then create multiple alignments from the similarities identified by the pairwise alignment. They both detect chimeric reads by finding regions covered by few alignments.

The differences between Sprai and HGAP are as follows. First, the seed reads of HGAP are a set of reads longer than a threshold given by the user, whereas Sprai takes an arbitrary set of reads and chooses error-corrected reads up to the user-specified depth (20× by default, the same as the target depth for seed reads in HGAP). This strategy is particularly useful when the proportion of chimeric and low-quality reads, which is usually unknown prior to error correction, is relatively high. Second, Sprai finds similarities between raw reads using NCBI BLAST+ [3], whereas HGAP uses BLASR [4]. BLAST+ does not create a search index in memory, making Sprai scalable to thousands of CPU cores as long as the bandwidth of the file system is scalable. BLASR uses a 32-bit integer array for its search index, so that we have to split input raw reads into smaller chunks, and therefore, we need an additional step to merge results, a step that is not necessary for BLAST+. Third, Sprai uses ReAligner [5] for multiply aligning raw PacBio reads, whereas HGAP uses PGDAGCon [2]. Fourth, HGAP avoids using repetitive alignments for consensus sequences by limiting the maximum number of alignments computed for each raw read, whereas Sprai limits the number of alignments used for consensus generation for each position on a seed read.

Performance benchmarks of Sprai are shown below.

Performance benchmarks of Sprai using the six bacterial genomes in the previous study [6].

Bacterial genome	*Escherichia coli* K12	*Escherichia coli* O157:H7	*Bibersteinia trehalosi*	*Mannheimia haemolytica*	*Francisella tularensis*	*Salmonella enterica*
Number of scaffolds	1	20	1	1	1	5
Total bases (Mb)	4.64	5.60	2.41	2.74	1.87	4.92
Max length (Mb)	4.64	3.83	2.41	2.74	1.87	3.10
N50 contig length	4.64	3.83	2.41	2.74	1.87	3.10

1. Myers EW, Sutton GG, Delcher AL, Dew IM, Fasulo DP, Flanigan MJ, Kravitz SA, Mobarry CM, Reinert KH, Remington KA, Anson EL, Bolanos RA, Chou HH, Jordan CM, Halpern AL, Lonardi S, Beasley EM, Brandon RC, Chen L, Dunn PJ, Lai Z, Liang Y, Nusskern DR, Zhan M, Zhang Q, Zheng X, Rubin GM, Adams MD, Venter JC: **A whole-genome assembly of Drosophila.** *Science* 2000, **287**:2196-2204.
2. Chin C-S, Alexander D, Marks P, Klammer A, Drake J, Heiner C, Clum A, Copeland A, Huddleston J, Eichler E, Turner S, Korlach J: **Nonhybrid, finished microbial genome assemblies from long-read SMRT sequencing data**. *Nat Methods* 2013:1-9.
3. Camacho C, Coulouris G, Avagyan V, Ma N, Papadopoulos J, Bealer K, Madden TL: **BLAST+: architecture and applications**. *BMC Bioinformatics* 2009, **10**:421.
4. Chaisson M, Biosciences P, Tesler G, California U: **Mapping single molecule sequencing reads using basic local alignment with successive refinement (BLASR): application and theory**. *BMC Bioinformatics* 2012, **13**(Methodology article):238.
5. Anson EL, Myers EW: **ReAligner: A Program for Refining DNA Sequence Multi-Alignments.** *JCB* 1997, **4**:369-383.
6. Koren S, Harhay GP, Smith TP, Bono JL, Harhay DM, McVey SD, Radune D, Bergman NH, Phillippy AM: **Reducing assembly complexity of microbial genomes with single-molecule sequencing.** *Genome Biol* 2013, **14**:R101-116.
