# Supplementary material for: Performance comparison of second- and third-generation sequencers using a bacterial genome with two chromosomes
Source: BMC Genomics. 2014 Aug 21;15(1):699. doi: 10.1186/1471-2164-15-699 (PMC4159541; doi:10.1186/1471-2164-15-699)
Supplement: Supplementary file 8 — Additional file 8: Table S3: Comparison between Sprai and HGAP assembly. The number of mismatches was calculated using QUAST v.2.3 [23]. (PDF 46 KB) [file 12864_2014_6410_MOESM8_ESM.pdf]

Additional file 8: Table S 3: Comparison between Sprai and HGAP assembly

**Assembly Statistics**	**HGAP**	**Sprai**	**Sprai (>1M bp)**
Number of contigs	31	31	2
Total bases	5172620	5298335	5164098
Max length	1745449	3288561	3288561
N50 contig length	552205	3288561	3288561
Number of mismatches	248	389	157
Number of indels	447	715	698
Indels length	559	818	794
Number of mismatches per 100 kbp	4.9	7.5	3.0
Number of indels per 100 kbp	8.8	13.8	13.5
Genome coverage (%)	98.744	99.999	99.848
Duplication ratio	1.034	1.032	1.007
